# Change in the Physiological and Biochemical Aspects of Tomato Caused by Infestation by Cryptic Species of *Bemisia tabaci* MED and MEAM1

**DOI:** 10.3390/insects12121105

**Published:** 2021-12-10

**Authors:** Claudia Aparecida de Lima Toledo, Franciely da Silva Ponce, Moisés Daniel Oliveira, Eduardo Santana Aires, Santino Seabra Júnior, Giuseppina Pace Pereira Lima, Regiane Cristina de Oliveira

**Affiliations:** 1 Department of Horticulture, São Paulo State University (UNESP), Botucatu 18600-950, Brazil; franciely.ponce@unesp.br (F.d.S.P.); e.aires@unesp.br (E.S.A.); 2 Crop Protection Department, São Paulo State University (UNESP), Botucatu 18600-950, Brazil; moisesdanielmdo@gmail.com (M.D.O.); regiane.cristina-oliveira@unesp.br (R.C.d.O.); 3 Department of Agronomy, State University of Mato Grosso, Nova Mutum 78450-000, Brazil; santinoseabra@hotmail.com; 4 Department of Chemistry and Biochemistry, Institute of Biosciences, Sao Paulo State University (UNESP), Botucatu 18618-970, Brazil; gpplima@ibb.unesp.br

**Keywords:** enzymes, insect–plant relationships, photosynthesis, *Solanum esculentum*

## Abstract

**Simple Summary:**

*Bemisia tabaci* is a polyphagous pest with cryptic species that infest various agricultural crops worldwide. Among the cryptic species, MED and MEAM1 are the most invasive, causing large production losses due to the feeding and transmission of the virus. We aimed to analyze the influence of these insects on the physiology and biochemistry of tomato. We found that the cryptic species MED caused considerable reduction in CO_2_ assimilation rates, stomatal conductance, and instantaneous carboxylation efficiency. Furthermore, plants infested with MED showed high activity of the enzymes peroxidase and polyphenol oxidase, which are considered expressions of plant defense. In contrast, plants infested with MEAM1 showed low peroxidase activity, which may result in a less lignified feeding place.

**Abstract:**

Infestation by *Bemisia tabaci* (Gennadius) (Hemiptera: Aleyrodidae) causes damage to tomatoes with production losses of up to 100%, affecting the physiological and biochemical aspects of host plants. The objective of this study was to analyze the influence of infestation of cryptic species of *B. tabaci* MED and MEAM1 on the physiological and biochemical aspects of tomato. Tomato plants ‘Santa Adélia Super’ infested with *B. tabaci* (MED and MEAM1), and non-infested plants were evaluated for differences in gas exchange, chlorophyll - a fluorescence of photosystem II (PSII), and biochemical factors (total phenols, total flavonoids, superoxide dismutase—SOD, peroxidase—POD, and polyphenol oxidase—PPO). Plants infested with *B. tabaci* MED showed low rates of CO_2_ assimilation and stomatal conductance of 55% and 52%, respectively. The instantaneous carboxylation efficiency was reduced by 40% in MED and by 60% in MEAM1 compared to the control. Regarding biochemical aspects, plants infested by MED cryptic species showed high activity of POD and PPO enzymes and total phenol content during the second and third instars when compared to control plants. Our results indicate that *B. tabaci* MED infestation in tomato plants had a greater influence than *B. tabaci* MEAM1 infestation on physiological parameters (CO_2_ assimilation rate (A), stomatal conductance (*gs*), and apparent carboxylation efficiency (A/Ci)) and caused increased activity of POD and PPO enzymes, indicating plant resistance to attack. In contrast, *B. tabaci* MEAM1 caused a reduction in POD enzyme activity, favoring offspring performance.

## 1. Introduction

Tomato (*Solanum esculentum* L.) is the second most important vegetable crop in the world because of its production and consumption. The main risk for tomatoes is pest in-festation, which can cause losses of up to 100% of production [1,2].

The whitefly, *Bemisia tabaci* (Gennadius) (Hemiptera: Aleyrodidae), is one of the larg-est phytosanitary challenges both in Brazil and globally, infesting crops in 60 countries and colonizing more than 600 plant species. *B. tabaci* is a complex cryptic group, with 44 sister species described, of which MED and MEAM1 are the most invasive [3]. Differentia-tion between these species is based on biochemical markers, choice of host plants, suscep-tibility to insecticides, ability to cause disturbances in the host plant, virus transmission, and endosymbiont types [4,5,6,7].

Losses caused by *B. tabaci* are related to direct damage, which is caused by the feeding process through the suction of phloem sap and injection of toxins. In addition, indirect damage occurs through viral transmission [8,9].

Plants perceive attacks by insect pests through compounds released in saliva, known as elicitors, and cause depolarization of the plasma membrane, followed by an increase in [Ca^2+^]_cyt_ and production of reactive oxygen species (ROS). The presence of Ca^2+^ in the cyto-sol activates calcium-dependent protein kinases and mitogen-activated protein kinases (MAPK) [10,11].

In response to insect attacks, plants can use induced or constitutive resistance. In-duced resistance makes the host plant less attractive and nutritious to pest insects, trig-gering plant-wide (systemic) responses. In addition, induced resistance can alert neighboring plants to insect attacks. Constitutive defense, such as trichomes, is present regardless of external stimuli [12,13].

The ROS are generated through the plant’s immune response, mediating the interac-tions of constitutive and induced defenses, resulting in phytohormone activation. Howev-er, at high concentrations, they degrade the structures of proteins, DNA, and other orga-nelles. The degradation of ROS occurs through two systems, enzymatic and non-enzymatic, the enzymatic being through antioxidant enzymes (superoxide dismutase (SOD), catalase, and peroxidase) that participate in several biochemical reactions in plants [14,15,16]. SOD catalyzes the dismutation reaction of superoxide (O_2_^-^*) into H_2_O_2_, and peroxidase (POD) and catalase (CAT) act on the peroxide to reduce it [12].

Peroxidase (POD) catalyzes the formation of lignins and oxidative phenols, which help to strengthen the cell structure. In contrast, Catalase (CAT) provides increased resistance to the cell wall and acts as a signal in the induction of defense genes [17]. In addition to antioxidant enzymes, there are defense enzymes such as polyphenol oxidase (PPO), which oxidize phenols into quinones and interact with the amino acid nucleotide side chain, causing the crosslinking of proteins that become unavailable to insects [18]. Phenylalanine ammonia lyase (PAL) is an enzyme involved in the biosynthesis of phytoalexins and lignins, which increases plant resistance. Furthermore, the increase in PAL levels leads to the accumulation of phenolic compounds, as this enzyme is involved in the synthesis of phenolic compounds [19]. The enzymes PPO, SOD, and CAT are involved in plant resistance to attack by herbivorous insects; therefore, the greater their activity in plants, the greater is the level of plant resistance to insect attack [20].

In Brazil, the invasion of MED in 2014 made it difficult to manage whitefly in crop-ping systems, as whitefly control is performed with the use of synthetic insecticides, and these have dosage rate on the package insert directed to *B. tabaci* MEAM1. Furthermore, identification of cryptic species requires molecular analysis techniques. The species *B. tabaci* MED and MEAM1 differ in host plant choice and insecticide susceptibility [7,21,22], where the use of insecticides to control the whitefly is considered one of the main factors related to the displacement of MEAM1 by MED in cultivated areas [23]. It is also known that, in Brazil, MED has an increased preference for sweet pepper, which is demonstrated by field collections in São Paulo, with only MED infestation in sweet pepper plants. In tomatoes, there was a proportion of both species in different cropping systems [24]. In a competitiveness study in tomato, MEAM1 displaced MED in insecticide-free environments after four generations [25].

In this sense, because of the differences between the cryptic species regarding the choice of the host plant, the hypothesis in this study is that infestation by cryptic species of *B. tabaci* (MED and MEAM1) shows different damage characteristics (physiological and biochemical) in the host plants, which could provide insights to explain the lowered preference of MED for tomato. Thus, the objective was to analyze the influence of feeding by cryptic species of *B. tabaci* MED and MEAM1 on the physiological and biochemical aspects of tomato.

## 2. Material and Methods

### 2.1. Identification of Cryptic Whitefly Species

Molecular analysis was performed by extracting total nucleic acids from individual whiteflies using the modified chelex method [26]. Individual samples of DNA were amplified by polymerase chain reaction (PCR) using the primer pair Bem23: Bem23F (5′-CGGAGCTTGCGCCTTAGTC-3′) and Bem23R (5′-CGGCTTTATCATAGCTCTCGT-3′) [27], which differentiated the MED and MEAM1 species based on a microsatellite locus [28]. To confirm the MED species by PCR, the primer pair 2195Bt (5′-TGRTTTTTTTGGTCATCCRGAAGT-3′) and C012/Bt-sh2 (5′-TTTACTGCACTTTCTGCC-3′) were used [29]. All digested DNA was analyzed by agarose gel electrophoresis (1.8%), stained with ethidium bromide, and visualized by UV transillumination [24].

### 2.2. B. tabaci MEAM1 and MED Mass Rearing

The virus-free cryptic species *B. tabaci* MED and MEAM1 were obtained from the col-lection held by the Research Group on Integrated Pest Management in Agriculture (AGRIMIP) at the School of Agronomy (FCA), São Paulo State University “Júlio de Mesquita Filho” (UNESP), Campus of Botucatu. The insects were kept and raised in an air-conditioned room (26 ± 2 °C, photophase of 14 h, and RH of 70 ± 10). Kale (*Brassica oleracea* var. *Acephala* L.) was used as the host plant for *B. tabaci* MEAM1, and the hybrid sweet pepper variety (*Capsicum annuum* L.) Magali R was used as the host for *B. tabaci* MED.

Host plants were grown in 750 mL pots containing Carolina Soil^®^ (Pardinho, SP, Brazil) commercial substrate, irrigated with 50 mL of H2O/day, and fertilized as required by the culture. Plants for insect multiplication were replaced every 30 days, immediately after the emergence of adult insects.

### 2.3. Treatments and Experimental Design

The experimental design was randomized blocks, consisting of 3 treatments and 10 repetitions, totaling 30 plots, with 3 plants per plot. The effects on plants infested with *B. tabaci* MED, *B. tabaci* MEAM1, and non-infested plants (control) were analyzed.

### 2.4. Description of the Site and Experiment Implementation and Conduction

The study was conducted at the School of Agronomy, FCA/São Paulo State Univer-sity “Júlio de Mesquita Filho”, UNESP, Campus of Botucatu, located at an altitude of 800 m above sea level, with coordinates 22°52′20″ S and 48°26′37″ W. The weather condi-tions of this region are warm temperate (mesothermal) humid (Cfa) (Koppen), with an average temperature in the hottest month above 22 °C.

The experiment was conducted in an arc-shaped protected environment (6 m × 21 m × 3 m, W × L × H), covered with a 150 µm polyethylene film, and side windows with 50% shading screens.

Santa Adélia Super (Topseed^®^) (Santo Antônio de Posse, SP, Brazil) tomato seedlings were sown in a 200 mL container, filled with commercial substrate (Carolina Soil^®^), and protected with voile fabric to avoid infestation with insects. After 30 days of sowing, the tomato seedlings were transplanted in 10 L pots filled with soil, sand, and manure in a ratio of 1:1:1. The spacing between the plants was 1 × 0.4 m. At planting, the following were applied: 60 kg·h^−1^ of N (urea), 300 kg·h^−1^ of P_2_O_5_ (simple superphosphate), and 100 kg·h^−1^ of K_2_O potassium chloride (KCl), and the covering treatment was carried out at 200 kg·h^−1^ of N and 120 kg·h^−1^ of K_2_O. Drip irrigation was applied, with a distance of 0.2 m between drippers.

The plants were kept throughout the experiment in individual cylindrical cages (D: 0.6 m, H: 1.5 m), consisting of galvanized wire (n.18, 1.24 mm), wrapped in white agrotextile fabric (17 g m^2^). The luminosity inside the cultivation cages was 27,200 lux.

The plants were infested at 33 DAS (days after sowing), with a manual aspirator, when the plants had 5–6 defined leaves. The procedure consisted of removing adults from an infested host plant and transferring them to the tomato plant. The insects remained on the plant for four days, to produce an infestation; then, the adult insects were removed, and the plants were monitored to follow the stages of development of the insects.

### 2.5. Variables Analyzed

#### 2.5.1. Analysis of the Physiological Aspects of Tomato

Gas exchange evaluations were performed using an infrared gas analyzer IRGA (In-fra-Red Gas Analyzer, model Li-6400; LI-COR Biosciences, Lincoln, NE, USA) at 34 days after plant transplantation, when the *B. tabaci* nymphs reached the third stage of development. The measurements were made on a sunny day, from 07:30 to 09:00, at an ambient temperature of 20 °C. Gas exchange was analyzed using CO_2_ assimilation rate parameters (A, μmol CO_2_ m^−2^s^−1^), transpiration rate (E, mol H_2_O m^−2^s^−1^), stomatal conductance (*gs*, mol m^−2^s^−1^), and internal CO_2_ concentration in the leaf (*Ci*, μmol CO_2_ mol^−1^). The water use efficiency (WUE, μmol CO_2_ [mmol H_2_O]^−1^) was determined from the relationship between CO_2_ assimilation and transpiration, and the apparent carboxylation efficiency (A/Ci) was established based on the CO_2_ assimilation (A) and the internal concentration of CO_2_ in the leaf (*Ci*) [13,29].

For chlorophyll *a* fluorescence, measurements were made on the third expanded leaf, counted from the apex of the plant, using a portable fluorometer coupled to the IRGA, in which the maximum fluorescence (Fm’) and minimum fluorescence (Fo’) were quantified under artificial light. Based on previous data, the maximum quantum efficiency of PSII (Fv/Fm), PSII maximum efficiency (Fv’/Fm’), coefficient of photochemical quenching (qP), non-photochemical extinction coefficient (NPQ), and apparent electron transport rate (ETR) were determined [30,31].

#### 2.5.2. Biochemical Analyses in Tomato

For biochemical analyses, expanded leaves, with no signs of senescence, were collected from the upper third of the plant. One leaf per plant was collected in each whitefly development phase (oviposition and second, third, and fourth instars), washed, placed in transparent plastic bags, identified, and wrapped in aluminum foil to prevent material degradation by light. The leaves were then subjected to freezing with liquid nitrogen for later maceration, placed in 15 g containers, and stored in a freezer at −20 °C.

The extraction used for the enzymes POD and PPO consisted of weighing 300 mg of the fresh sample, homogenized with 8 mL of 0.2 M potassium phosphate buffer solution (pH 6.7), and centrifuged 6000× *g* at 5 °C for 15 min (model Mikro 220R; Hettich). The POD (EC 1.11.1.7) activity was determined according to the method described by Lima et al. [32]. The H_2_O_2_ and phenol solutions were added to the crude extract, and the samples were placed in a water bath at 30 °C for 5 min. Readings were performed at 505 nm ab-sorbance, and POD activities were expressed as µmol H_2_O_2_ decomposed·min^−1^ g^−1^ FW^−1^. The activity of PPO (EC 1.10.3.1) was determined according to the methodology of Kar and Mishra [33], using catechol as a substrate. For the reaction, 300 µL of enzymatic extract and 1.85 mL of 0.1M catechol were used. Afterwards, the tubes were placed in a water bath (30 °C) for 30 min. Absorbance was measured at 395 nm, and the results expressed in µmol catechol were transformed into min^−1^g^−1^ FW^−1^.

The extract used for SOD (EC 1.11.1.6) consisted of 300 mg of plant material macer-ated in liquid nitrogen and homogenized with 5 mL of 100 mmol potassium phosphate L-1 (pH 7.5), supplemented with tetrasodium ethylenediaminetetraacetic acid (EDTA), di-thiothreitol (DTT), and polyvinylpyrrolidone (PVPP). After centrifugation (6000× *g* at 4 °C) for 20 min, the supernatant was collected and stored in an amber bottle in a freezer at −20 °C.

The SOD content was determined using the methodology proposed by Sun et al. [34], with modifications. The reaction process consisted of pipetting 2 mL sodium phosphate buffer (pH 7.8), 50 µL of the enzyme extract, 250 µL of tetrazolium blue nitro chloride (NBT), 200 µL of tetrasodium ethylenediaminetetraacetic acid, 250 µL of methionine, and 250 µL of riboflavin. The reaction took place at 25 °C for 10 min in a chamber containing fluorescent lamps (20 W). The reaction was stopped when the lamps were turned off [35], and the absorbance was read at 560 nm. The enzyme content was determined based on the inhibition of the reduction of tetrazolium blue nitro chloride, with the need to inhibit 50% of the photoreduction defined as one unit of activity, and the content of SOD was expressed as U of activity g^−1^ FW^−1^.

The total phenol content was determined spectrophotometrically using the Folin–Ciocalteu reagent [36]. Fresh leaf samples (50 mg) were extracted in 4 mL of 80% methanol, acidified with 1% acetic acid (80/19/1, v/v/v), homogenized, and placed in an ultrasonic bath for 20 min (model Q3.0/40A; Eco-Sonics). Then, the samples were centrifuged at 5000× *g* at 4 °C (model Mikro 220R; Hettich), and the supernatant was removed and retained. The extraction process was repeated, and the supernatants were pooled in an amber container. Absorbance was analyzed at 725 nm, and the total phenol content was expressed as gallic acid equivalent 100 g^−1^ of fresh weight (mg GAE 100g^−1^ FW^−1^).

The total flavonoid content was determined using the methodology described by Popova et al. [37], with adaptations. Fresh leaf samples (100 mg) were extracted in methanol and placed in an ultrasonic bath (model Q3.0/40A; Eco-Sonics) for 30 min. In the samples, 1 mL of 5% aluminum chloride was added, homogenized, and placed in the dark. Subsequently, the reaction was centrifuged at 6000× *g* (model Mikro 220R; Hettich). The results were expressed as mg quercetin per 100 g^−1^ of fresh weight (mg QE 100g^−1^ FW^−1^).

### 2.6. Statistical Analysis

The results were subjected to exploratory analyses to assess the normality [38] and homogeneity of the data [39]. Analysis of variance (ANOVA) was performed and differ-ences were compared (Control, MED, and MEAM1) using the Tukey’s test at a significance level of 5% using the AGROESTAT software [40]. The variables analyzed were A, Gs, A/Ci, Fv’/Fm’, non-photochemical extinction coefficient, SOD, POD, PPO, and total phenols and flavonoids.

## 3. Results

### 3.1. Physiological Aspects of Tomato

Tomato plants infested with *B. tabaci* (MED and MEAM1) showed changes in physi-ological parameters such as A, Gs, A/Ci, Fv’/Fm’, and non-photochemical extinction coef-ficient of PSII (Table 1) when compared to control plants.

Tomato plants infested with *B. tabaci* MED had a reduced rate of CO_2_ assimilation (liquid photosynthesis) (A) by 55.5% (7.09 µmol CO_2_m^−2^s^−1^) compared to control plants (*f* 26.73, 20, *p* < 0.0001) (Table 1). However, this trend was not observed in plants infested with *B. tabaci* MEAM1.

Plants infested with *B. tabaci* MED had lower *gs* (0.13 mol m^−2^ s^−1^) than plants infested with *B. tabaci* MEAM1, which had *gs* of 0.40 molm^−2^s^−1^. However, there was no difference in *gs* when comparing infested and non-infested plants (*f* 13.05, 20, *p* < 0.001) (Table 1).

The A/Ci decreased by 40% (0.03 ± 0.01) and 60% (0.02 ± 0.01) in tomato plants in-fested with *B. tabaci* MED and MEAM1, respectively (*f* 25.95, 20, *p* < 0.0001) (Table 1).

Plants infested with *B. tabaci* MED showed a reduction of approximately 11% in Fvʹ/Fmʹ when compared to plants infested with MEAM1 (*f* 6.14, 20, *p* < 0.0145). This pa-rameter allows the analysis of the capacity of chlorophylls to absorb photons.

Tomatoes infested by the cryptic species MED showed a higher NPQ of 12% (1.56 ± 0.21) than control plants (1.39 ± 0.25) (*f* 8.57, 20, *p* < 0.0049) (Table 1). This increase in NPQ can prevent damage caused by stress due to excess energy in the photosynthetic appa-ratus. In this sense, the increase in NPQ induced by the feeding of *B. tabaci* can reduce the number of electrons flowing through photosynthesis, that is, reducing the stress due to excess light in photosystem II.

Plants infested with MED showed increased damage to the photosynthetic apparatus, causing reduced photosynthesis and stomatal conductance, and consequently, low instant carboxylation efficiency. Consequently, rubisco activity has an increased influence on the production of photoassimilates and biomass production.

### 3.2. Biochemical Aspects of Tomato

The tomato plants infested with *B. tabaci* (MED and MEAM1) and not infested (con-trol) showed biochemical differences in the following parameters: POD, PPO, total phenols, and flavonoids in the analyzed instars, but did not differ in activity of the SOD enzyme (Table 2).

Plants infested with *B. tabaci* (MED and MEAM1) influenced POD activity at all developmental stages of the evaluated insects (Table 2). During oviposition, MEAM1 infested plants showed lower POD activity by 46% and 52% when compared to control and MED plants, respectively (*f* 82.20, 8, *p* < 0.0001).

In the second instar, plants infested with *B. tabaci* MED showed higher enzymatic activity (POD) (*f* 23.78, 8, *p* < 0.0014) than the control and MEAM1 infected plants, at rates of 53% and 30%, respectively. There was no statistically significant difference between plants not infested (control) and those infested with MEAM1 (Table 2).

For the third instar, the plants infested by the cryptic species of *B. tabaci* (MED and MEAM1) (*f* 34.23, 8, *p* < 0.0005) showed no statistically significant difference between them, the only difference being between infested and non-infested plants. However, plants attacked by *B. tabaci* MEAM1 exhibited 42% higher POD activity at the fourth instar of insect development than control plants, and 29% higher activity than plants infested with MED at this stage (*f* 42.89, 8, *p* < 0.0003) (Table 2).

During oviposition, plants infested with *B. tabaci* (MED and MEAM1) showed higher PPO enzyme activity than control plants (*f* 192.63, 8, *p* < 0.0001); however, the cryptic species MEAM1 had higher enzyme activity than the control and MED at approximately 55% (173.38 µmol catechol min^−1^·g^−1^ FW^−1^) and 38% (239.49 µmol catechol min^−1^·g^−1^ FW^−1^, respectively).

In the second and third instars, plants infested with MED had higher PPO enzyme activity than those in the other treatments. In the second instar, tomato plants showed higher PPO activity, approximately 77% and 30% higher than the control and MEAM1 infested plants (*f* 65.95, 8, *p* < 0.0001). In the third instar, plants infested with MED had higher PPO activity than the control and MEAM1 by 16% and 67%, respectively (*f* 466.21, 8, *p* < 0.0001).

During oviposition, infestation by the cryptic species resulted in greater production of total phenols than in control tomato plants, with a 19% and 32% increase for MEAM1 and MED (*f* 38.90, 8, *p* < 0.0004), respectively.

In the second and third instar, tomato plants infested with MED had a higher pro-duction of total phenols. In the second instar, there was no statistically significant difference between the two cryptic species analyzed, but tomato plants infested with MED had higher total phenol production than control plants (*f* 30.54, 8, *p* < 0.0007) (Table 2). In the third instar, MED infested plants had higher levels than the control and MEAM1 plants by 23% and 34% (*f* 80.22, 8, *p* < 0.0001), respectively.

In the fourth instar, the tomato infested with MED had a lower content of total phenols compared to the control and plants infested with MEAM1 (*f* 9.52, 8, *p* < 0.0018) (Table 2). This may be related to the reduction of food in this phase [41].

As for the flavonoid content in the infested and control plants, there was a statistically significant difference only in the oviposition and fourth-instar nymph stages (Table 2). The plants attacked by MED showed higher levels of flavonoids than the control plants during oviposition; however, there was no difference between those infested with the cryptic species (*f* 6.49, 8, *p* < 0.0316). In the fourth instar, the plants infested with MEAM1 had higher levels of flavonoids than those in the other treatments (*f* 21.58, 8, *p* < 0.0018) (Table 2).

## 4. Discussion

### 4.1. Physiological Aspects of Tomato

Photosynthesis is indirectly affected by factors such as availability of water, CO_2_, light and temperature, these factors can directly interfere with the opening of the stomatal cleft. Tomato plants infested with *B. tabaci* (MED and MEAM1) presented interference in physiological parameters. However, between the cryptic species evaluated, the plants infested by MED had a greater reduction in CO_2_ assimilation, stomatal conductance, instantaneous carboxylation efficiency, and maximum PSII efficiency. It is important to note that these parameters are interdependent; thus, interference in one parameter can reflect on others.

The *gs* was one of the parameters affected by the infestation of *B. tabaci* MED. Inter-ference in *gs* directly affects photosynthesis, as a reduction in gs causes resistance to CO_2_ uptake, and consequently lower CO_2_ concentration in the leaf mesophile, resulting in lower availability for chloroplasts and, therefore, lower production of photoassimilates [42,43,44].

When *gs* is high, there is an increase in the CO_2_ assimilation rate depending on the use of internal carbon to maintain the variation of the chemical CO_2_ gradient, which al-lows entry into the leaf. Plants with lower *gs* have a lower CO_2_ assimilation rate because the opening of the stomatal gap allows the entry of CO_2_ into the leaf mesophyll and a re-duction in water loss. Thus, the greater the limitation of *gs*, the lower is the carbon dioxide content in the leaf mesophyll, causing less substrate for the photosynthetic process, influ-encing the production of carbohydrates, and interfering with the development of plants. Reduced *gs* was also observed in citrus infested by *Aleurocanthus woglumi* (Ashby, 1915) (Hemiptera: Aleyrodidae) [45].

Changes in *gs* are the main factors that affect the photosynthetic performance of plants, as they directly interfere with the instantaneous efficiency of carboxylation (A/Ci). There is a close relationship between *gs* and *Ci*, as they increase simultaneously. Furthermore, the instantaneous efficiency of carboxylation is directly related to the assimilation of CO_2_ and its intercellular concentration; therefore, the reduction in A/Ci is owing to the lower rate of CO_2_ assimilation and, consequently, to the lower internal concentration of carbon dioxide in the leaf mesophile [46,47].

Plants infested with *B. tabaci* MED showed a reduction in the maximum efficiency of PSII (Fv’/Fm’), which is a parameter that allows the analysis of the capacity of chlorophylls to absorb photons. Furthermore, it reveals the physiological state of chlorophylls, which can interrupt the ability to absorb photons, reduce the production of ATP and NAD, and affect photosynthesis [48,49].

MED infestation had a high NQP, a process that seeks to avoid damage to the photosynthetic apparatus. In this process, unused energy is dissipated in a nonradioactive manner, increasing the NPQ. This increase in NPQ can prevent damage caused by stress due to excess energy in the photosynthetic apparatus. In this sense, the increase in NPQ induced by the feeding of *B. tabaci* can reduce the number of electrons flowing through photosynthesis, that is, reducing the stress due to excess light in photosystem II [48,50,51,52].

Another important factor to be mentioned is the influence of *B. tabaci* on photosynthetic parameters, which is a result of indirect and direct damage during feeding. This is because feeding causes a reduction in plant vigor, and the sugar released by insects that feed on the phloem promotes the development of fungi of *Capnodium*, on the surface of plants, hindering physiological processes such as photosynthesis, respiration and transpiration of parts attacked, which can block photosynthetically active radiation, causing a reduction of up to 70% of CO_2_ assimilation [9,53]. In this study, plants infested with *B. tabaci* showed yellow spots on the leaves in places with nymphs. Furthermore, the presence of sugary exudates in the leaves was observed; however, little sooty mold was observed.

Plants infested with MED showed increased damage to the photosynthetic apparatus, causing reduced photosynthesis and stomatal conductance, and consequently, low instant carboxylation efficiency. Consequently, rubisco activity has an increased influence on the production of photoassimilates and biomass production.

### 4.2. Biochemical Aspects of Tomato

In the oviposition phase and second instar of *B. tabaci* MEAM1, the tomato showed reduced activity of the POD enzyme, which is an indicator of lower stress on the plants. This defense strategy against the tomato immune system is correlated with the secondary *Hamiltonella* endosymbionts, which cause the tomato to reduce the activity of POD enzymes, being a likely way to ensure that the offspring find the less lignified plant tissue, facilitating the insertion of the oral appliance [54].

Plants identify insect oviposition from exudates released during laying, through hypersensitivity responses, commonly necrosis, neoplasia, increase in reactive oxygen species and defense proteins. Plants can kill eggs by crushing them by cultivating new tissue at the laying site, or even killing them by synthesizing ovicidal substances [55]. These activities result in the death of insects yet at the egg stage [55,56].

The third instar stage of the cryptic species of *B. tabaci* (MED and MEAM1) is the most harmful because of the suction of the phloem sap, which causes greater activity of the POD enzyme in tomato. The increased activity of this enzyme, caused by pest pressure, makes the attractive tissue less attractive to insects because of the transformation of phe-nols into quinone, and its participation in lignin synthesis, resulting in stiffness of the attacked tissue. The trend of increasing POD activity was maintained for MEAM1 in the fourth instar [13,57].

As for the enzyme polyphenoloxidase (PPO), there was increased activity of this en-zyme in response to oviposition by *B. tabaci* MEAM1, and in the second and third instar of development of *B. tabaci* MED. The PPO enzyme is responsible for transforming phenolic compounds into reactive quinones, creating less attractive tissues for pests that feed on the tissue [12].

However, the greater PPO activity caused by *B. tabaci* MED demonstrated that tomato plants are tolerant to the attack by this cryptic species, as the greater the activity of the resistance enzymes POD and PPO, the greater was the defense of the plant against infestation by insects [18,58].

Although PPO and POD transform phenols into quinone, POD is also involved in the biosynthesis of lignin and suberin [57], and the increased concentration of these substances hardens the cell wall, making it difficult to insert the mouth stylet of insects. Plants with higher production of antioxidant and defense enzymes (SOD, POD, PPO, CAT, and PAL) are considered more resistant. This trend was observed in peanuts showing higher enzyme activity and total phenol content, resulting in cultivars resistant to *Helicoverpa armigera* (Hübner, 1809), *Spodoptera litura* (Fabricius, 1775) (Lepidoptera: Noctuidae), and *Empoasca kerri* (Pruthi, 1940) (Hemiptera: Cicadellidae) [14]. Castor bean infested by *Trialeurodes ricini* (Misra, 1924) (Hemiptera: Aleyrodidae) also showed increased enzymatic activity [59].

Total phenols are important metabolites in tomato defense against herbivorous in-sects. These compounds inhibit insect feeding through their ability to provide toxicity, bind to soluble proteins, form non-digestible compounds, and inactivate enzymes in the insect digestive tract, which can lead to death [60]. Thus, plants with high levels of phe-nolic compounds may cause higher insect mortality, deformation of nymphs, or changes in biological parameters. In this sense, it was found that plants infested with *B. tabaci* (MED and MEAM1) had higher production of total phenols during oviposition and the second instar. In the third instar, plants attacked by MED showed high production of total phenols. This increase during oviposition is probably directly related to the increased activity of PAL, the first enzyme in the phenylpropanoid pathway, which is involved in the biosynthesis of phenols and salicylic acid (SA). This result was observed in Brassica folwwoing oviposition by *Pieris brassicae* (Linnaeus, 1758) (Lepidoptera: Pieridae), in which there was an accumulation of SA and the genes (EDS1 and NPR1), responsible for regulating the activity of the PAL enzyme, causing greater production of SA and, consequently, greater production of phenolic compounds [19,20,61].

The high content of flavonoids in tomatoes attacked by *B. tabaci* MED during oviposition relates to the detection by plants of the presence of eggs on the leaf surface and the anticipated response to future feeding by hatched insects. The high content of flavonoids produced are linked to the fluidity and rigidity of the cell membrane. Low levels of these compounds facilitated the feeding of insects in these plants, and therefore constituted an increased preference for oviposition by females of *B. tabaci* [62,63]. However, in the fourth instar, the highest flavonoid content was observed for MEAM1.

Based on the data presented here, we suggest that tomato is more susceptible to damage caused by *B. tabaci* MEAM1, due to its ability to suppress plant defense mechanisms. During oviposition, the ability of MEAM1 to interfere with the activity of the POD enzyme allows the offspring to have fewer lignified sites for insertion of the oral appliance. However, *B. tabaci* MED does not manipulate tomato defense; rather, it allows the activation of defense enzymes, which consequently can reduce infestation and host plant preference. It is likely that the ability to manipulate the defense caused by *B. tabaci* MEAM1 is related to the adaptation of this insect-pest to tomatoes in Brazil due to the time of adaptation, since the first report of MEAM1 occurred in the 1990s; while MED was registered just two decades later, in 2014. During that time, MEAM1 was forced to improve its attack strategies against the tomato defenses, currently having an adaptive advantage over MED.

## 5. Conclusions

Tomato plants infested with cryptic species of *B. tabaci* MED showed greater interfer-ence with their physiological and biochemical parameters than *B. tabaci* MEAM1. *B. tabaci* MED caused greater interference in the CO_2_ assimilation rate, stomatal conductance, and apparent carboxylation efficiency.

In terms of biochemical responses, tomato plants infested with *B. tabaci* MEAM1 showed lower POD enzyme activity, which may result in less lignified places for insertion of the insects’ mouthparts, which may favor colonization by this cryptic species. As for MED, a shorter adaptation time may not have favored the development of strategies such as those presented by *B. tabaci* MEAM1.

## Figures and Tables

**Table 1:** Influence on physiological parameters caused by *Bemisia tabaci* (MED and MEAM1) in tomato.

Treatments	A	*gs*	A/Ci	Fv’/Fm’	NPQ
Control	15.93 ± 2.68 a	0.27 ± 0.07 ab	0.05 ± 0.01 a	0.57 ± 0.03 ab	1.39 ± 0.25 ab
MED	7.09 ± 1.71 b	0.13 ± 0.09 b	0.02 ± 0.01 c	0.53 ± 0.03 b	1.56 ± 0.21 a
MEAM1	12.60 ± 1.52 a	0.40 ± 0.10 a	0.03± 0.01 b	0.60 ± 0.04 a	1.06 ± 0.14 b

Means followed by the same letter in the column do not differ statistically by the Tukey’s test at 5% (*p* < 0.05). Subtitles: CO_2_ assimilation rate (A, µmol CO_2_m^−2^s^−1^), stomatal conductance (*gs,* mol m^−2^s^−1^], apparent carboxylation efficiency (A/Ci), maximum efficiency of PSII (Fv’/Fm’), non-photochemical extinction coefficient (NPQ).

**Table 2:** Superoxide dismutase, peroxidase, polyphenol oxidase, total phenols, and flavonoids (mean ± SD) in tomatoes infested by *Bemisia tabaci* MED and MEAM1 and non-infested tomatoes (control), at each instar of the insect.

Treatments		SOD	POD	PPO	Total Phenols	Flavonoids
0	Control	41.03 ± 0.13 a	0.15 ± 0.00 a	173.38 ± 21.79 c	126.55 ± 13.97 b	61.53 ± 1.25 b
	MED	40.99 ± 0.34 a	0.17 ± 0.01 a	239.49 ± 5.34 b	167.86 ± 7.83 a	73.68 ± 4.66 a
	MEAM1	40.97 ± 0.02 a	0.08 ±0.00 b	389.13 ± 5.34 a	150.81 ± 4.85 a	63.82 ± 5.87 ab
2	Control	41.47 ± 0.01 a	0.14 ± 0.02 b	106.76 ± 7.14 c	121.76 ± 13.97 b	53.62 ± 1.31 a
	MED	41.16 ± 0.28 a	0.30 ±0.04 a	467.13 ± 62.79 a	192.95 ± 7.83 a	57.51 ± 5.87 a
	MEAM1	40.97 ± 0.44 a	0.21 ± 0.00 b	323.91 ± 22.31 b	177.89 ± 3.21 a	59.90 ± 5.98 a
3	Control	41.16 ± 0.28 a	0.13 ± 0.00 b	685.32 ± 10.16b	164.45 ± 2.58 b	69.56 ± 3.38 a
	MED	41.11 ± 0.35 a	0.23 ± 0.03 a	823.70 ± 35.83 a	214.67 ± 11.98 a	69.09 ± 2.32 a
	MEAM1	41.12 ± 0.13 a	0.26 ± 0.01 a	270.09 ± 14.68 c	142.07 ± 2.16 c	68.61 ± 12.20 a
4	Control	41.18 ± 0.22 a	0.18 ± 0.01 b	376.68 ± 39.20 a	151.29 ±12.75 a	69.11 ± 1.69 b
	MED	41.08 ± 0.01 a	0.22 ± 0.00 b	309.58 ± 15.32 a	121.97 ±4.46 b	65.68 ± 0.78 c
	MEAM1	41.29 ± 0.11 a	0.31 ±0.03 a	299.62 ± 65.53 a	143.45 ±5.91 a	72.60 ± 1.23 a

Means followed by the same letter in the column do not differ statistically by the Tukey’s test at 5% (*p* *<* 0.05). Subtitles: 0—Oviposition, 2—second instar, 3—third instar, 4—fourth instar. Superoxide dismutase (SOD) (U of activity g^−1^ FW^−1^), peroxidase (POD) (µmol H_2_O_2_ min^−1^g^−1^FW^−1^), polyphenol oxidase (PPO) (µmol catechol min^−1^g^−1^FW^−1^), total phenols (mg of EAG 100 g^−1^ FW^−1^), flavonoids (mg of EQ 100 g^−1^ FW^−1^).
